# The Demographic and Clinical Characteristics, Prognostic Factors, and Survival Outcomes of Head and Neck Carcinosarcoma: A SEER Database Analysis

**DOI:** 10.3390/biomedicines12112556

**Published:** 2024-11-08

**Authors:** Wanting Hou, Ouying Yan, Hong Zhu

**Affiliations:** Division of Abdominal Tumor Multimodality Treatment, Cancer Center, West China Hospital, Sichuan University, Chengdu 610065, China; houwanting@wchscu.cn (W.H.); yanouying@stu.scu.edu.cn (O.Y.)

**Keywords:** head and neck carcinosarcoma (HNCS), surveillance, epidemiology, end results database (SEER database), cancer-specific survival (CSS), overall survival (OS), prognostic nomogram

## Abstract

Background: Head and neck carcinosarcoma (HNCS) is a rare and highly aggressive malignancy with limited research, resulting in an incomplete understanding of disease progression and a lack of reliable prognostic tools. This study aimed to retrospectively analyze the clinical characteristics and outcomes of HNCS patients using data from the Surveillance, Epidemiology, and End Results (SEER) database and to develop a nomogram to predict overall survival (OS) and cancer-specific survival (CSS). Methods: Patients diagnosed with HNCS from 1975 to 2020 were identified in the SEER database. Univariate and multivariate Cox regression analyses were conducted to identify independent prognostic indicators, with the optimal model selected using the minimal Akaike Information Criterion (AIC). The identified prognostic factors were incorporated into nomograms to predict OS and CSS. Model performance was assessed using the concordance index (C-index), area under the curve (AUC), calibration curves, and decision curve analysis (DCA). Survival curves were generated using Kaplan–Meier analysis and compared via the log-rank test. Results: A total of 152 HNCS patients were included, with 108 assigned to the training cohort and 44 to the validation cohort in a 7:3 ratio. Prognostic factors including age, primary tumor site, marital status, radiotherapy, chemotherapy, tumor size, pathological grade, and tumor stage were incorporated into the nomogram models. The models demonstrated strong predictive performance, with C-index values for OS and CSS of 0.757 and 0.779 in the training group, and 0.777 and 0.776 in the validation group, respectively. AUC values for predicting 3-, 5-, and 10-year OS were 0.662, 0.713, and 0.761, and for CSS the values were 0.726, 0.703, and 0.693. Kaplan–Meier analysis indicated significantly improved survival for patients with lower risk scores. The 3-, 5-, and 10-year OS rates for the entire cohort were 54.1%, 45.6%, and 35.1%, respectively, and the CSS rates were 62.9%, 57.5%, and 52.2%, respectively. Conclusions: This study provides validated nomograms for predicting OS and CSS in HNCS patients, offering a reliable tool to support clinical decision-making for this challenging malignancy. These nomograms enhance the ability to predict patient prognosis and personalize treatment strategies.

## 1. Introduction

Carcinosarcoma is a rare and highly aggressive malignancy characterized by the coexistence of both epithelial-derived carcinomatous and mesenchymal-derived sarcomatous components, presenting a unique challenge in understanding its pathogenesis and clinical behavior [1]. The precise histogenesis of carcinosarcoma remains unclear [2]; however, multiple theories have been proposed, with the monoclonal origin hypothesis gaining the most support. This theory posits that both carcinomatous and sarcomatous elements originate from totipotent stem cells capable of dual differentiation into mesenchymal and epithelial lineages [3,4].

Carcinosarcomas can arise in various anatomical sites, including the lungs, skin, digestive tract, and genitourinary and respiratory systems [2,5]. However, their occurrence in the head and neck is exceedingly rare. Evidence suggests that head and neck carcinosarcomas (HNCS) are more prevalent among older adults, though recent data indicate a potential trend toward younger age groups. Established risk factors, such as excessive alcohol consumption, smoking, and prior radiation exposure, are thought to contribute to HNCS development [6]. Notably, HNCS exhibits gender-specific patterns: men are more likely to present with tumors in the larynx and parotid glands, whereas women more commonly develop carcinosarcomas in the nasal sinuses.

Despite these observations, the literature on HNCS remains limited primarily to case reports, with few large-scale analyses that focus on survival outcomes or treatment efficacy. Consequently, there are no standardized treatment guidelines for HNCS, and clinicians lack a comprehensive understanding of its biological behavior and prognosis [7]. In this study, we utilized the SEER database to examine demographic and clinical characteristics, prognostic factors, and survival outcomes of patients with HNCS. Additionally, we developed nomograms to predict overall survival (OS) and cancer-specific survival (CSS), offering a practical tool for individualized prognosis assessment in HNCS patients.

## 2. Materials and Methods

### 2.1. Data Source and Patient Selection

Clinicopathological data for 152 patients with a first primary diagnosis of HNCS between 1975 and 2020 were extracted from the SEER database (www.seer.cancer.gov (accessed on 23 March 2024)). Cases were identified using the ICD-O-3 histological codes 8980/3 (carcinosarcoma, not otherwise specified) and 8981/3 (embryonal carcinosarcoma), with anatomical site codes C00–C14.8 and C30–C33.9.

The inclusion criteria were (a) histopathologically confirmed HNCS diagnosis and (b) age over 18 years. Patients were excluded if they had (a) incomplete clinical demographic data or (b) no follow-up information available.

The clinical variables collected included age, sex, ethnicity, year of diagnosis, primary tumor site, marital status, surgical treatment, radiotherapy, treatment sequence (surgery and radiotherapy), chemotherapy, tumor size, pathological grade, tumor stage, regional lymph node involvement, survival months, CSS, and OS.

### 2.2. Statistical Analysis

All statistical analyses were performed using R version 4.3.2 and SPSS version 17.0. OS was defined as the time from diagnosis to death from any cause, and CSS as the time from diagnosis to death specifically due to HNCS. Descriptive statistics were calculated for all clinical variables.

Eligible patients were randomly divided into training and validation cohorts at a 7:3 ratio using R’s random sampling function. The training cohort was used for model development, while the validation cohort assessed the model’s performance and generalizability. Chi-square tests were applied to examine differences in baseline variables between these cohorts, ensuring effective randomization.

Kaplan–Meier analysis was conducted to generate OS and CSS curves, with differences evaluated via the log-rank test. Univariate and multivariate Cox regression analyses were performed to identify independent prognostic factors for HNCS. Statistical significance was set at a *p*-value of <0.05.

### 2.3. Method for Constructing and Validating the Nomogram

Nomograms for predicting OS and CSS were developed and validated using the training and validation cohorts defined in Section 2.2. The optimal predictive model was selected based on the lowest Akaike Information Criterion (AIC) value, with AIC-identified prognostic factors incorporated into the nomogram models for OS and CSS [8]. To assess the accuracy of these nomograms, internal validation was performed with 1000 bootstrap resamples [9]. The concordance index (C-index) measured the model’s discriminative power, where values approaching 1.0 indicate higher predictive accuracy [10]. Calibration curves, featuring a data-derived line alongside a 45-degree reference line, were used to evaluate the model’s goodness of fit; close alignment between these lines reflected model accuracy [11]. Model performance was further assessed using receiver operating characteristic (ROC) curves and their area under the curve (AUC) values. Lastly, decision curve analysis (DCA) was conducted to evaluate the clinical utility of the model.

## 3. Results

### 3.1. Patient Clinicopathological Data

From the SEER database, 194 patients with HNCS diagnosed between 1975 and 2020 were initially identified, with 42 patients excluded based on the inclusion and exclusion criteria (see Supplementary Figure S1). The remaining 152 patients were randomly assigned to a training cohort (*n* = 108) and a validation cohort (*n* = 44), with detailed demographic and clinical characteristics presented in Table 1.

The division into training and validation cohorts aims to strengthen the robustness of the prognostic model. The training cohort allows for identification of key prognostic factors and the development of the nomogram, optimizing the model to fit the specific characteristics of our patient population. The validation cohort, in turn, provides an independent dataset to test the model’s performance, reducing the risk of overfitting and ensuring reliability of predictions. This two-stage approach supports the creation of a model that is both accurate and generalizable for clinical application.

Among the 152 patients, 96 (63.2%) were male and 56 (36.8%) were female, with a majority (91, 59.9%) diagnosed at an age over 60 years. Analysis of age trends from 1975 to 2020 reveals a gradual increase in the average age at diagnosis (Supplementary Figure S2). Most patients were white (124, 81.6%). The most common primary tumor sites included the salivary glands (42.8%), followed by the larynx (21.1%), nose/nasal cavity/middle ear (15.1%), oral cavity (15.1%), hypopharynx (3.3%), and nasopharynx (2.6%).

Regarding disease stage, regional disease was most common (36.2%), followed by localized disease (34.2%) and distant disease (11.2%). Treatment patterns varied, with only 33 patients (21.7%) receiving chemotherapy, while 127 (83.6%) underwent surgery, and 105 (69.1%) received radiation therapy (Table 1).

### 3.2. Identification of Independent Prognostic Factors for HNCS Using Univariate and Multivariate Analyses

In the training cohort, univariate analysis identified several factors significantly associated with OS, including age at diagnosis, primary tumor site, marital status, and tumor stage (*p* < 0.05). Additionally, primary tumor site, surgical treatment, and tumor stage were significantly correlated with CSS (*p* < 0.05).

To further delineate independent prognostic factors, a multivariate analysis was conducted. The analysis revealed that age over 60 years, primary tumor site in the oral cavity, single marital status, and distant tumor stage were independent risk factors for OS (*p* < 0.05; Table 2). For CSS, primary tumor site in the oral cavity and distant tumor stage emerged as independent risk factors (*p* < 0.05; Table 3).

### 3.3. Survival Statistics

**Figure 1:**
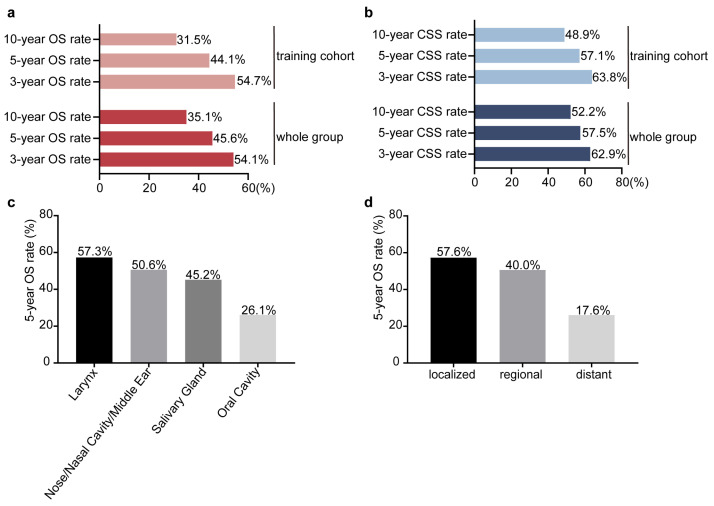
Survival outcomes for HNCS by cohort (**a**,**b**), tumor site (**c**), and disease stage (**d**).

Survival data were available for all 152 patients, with 102 (67.1%) recorded deaths and a median survival time of 38 months. The median survival time in the training and validation cohorts was 46 and 38 months, respectively. For the entire cohort, the 3-, 5-, and 10-year OS rates were 54.1%, 45.6%, and 35.1%, respectively, while CSS rates were 62.9%, 57.5%, and 52.2% (Figure 1a). In the training cohort, the 3-, 5-, and 10-year OS rates were 54.7%, 44.4%, and 31.5%, and the CSS rates were 63.8%, 57.1%, and 48.9% (Figure 1b).

By primary tumor site, patients with laryngeal tumors had the highest 5-year OS rate (57.3%), followed by those with tumors in the nose, nasal cavity, and middle ear (50.6%), salivary gland (45.2%), and oral cavity (26.1%) (Figure 1c). The 5-year OS rate also varied significantly by disease stage, with patients having localized disease showing a higher 5-year OS (57.6%) compared to those with regional (40.0%) or distant disease (17.6%) (Figure 1d).

### 3.4. Construction and Validation of Nomograms

**Figure 2:**
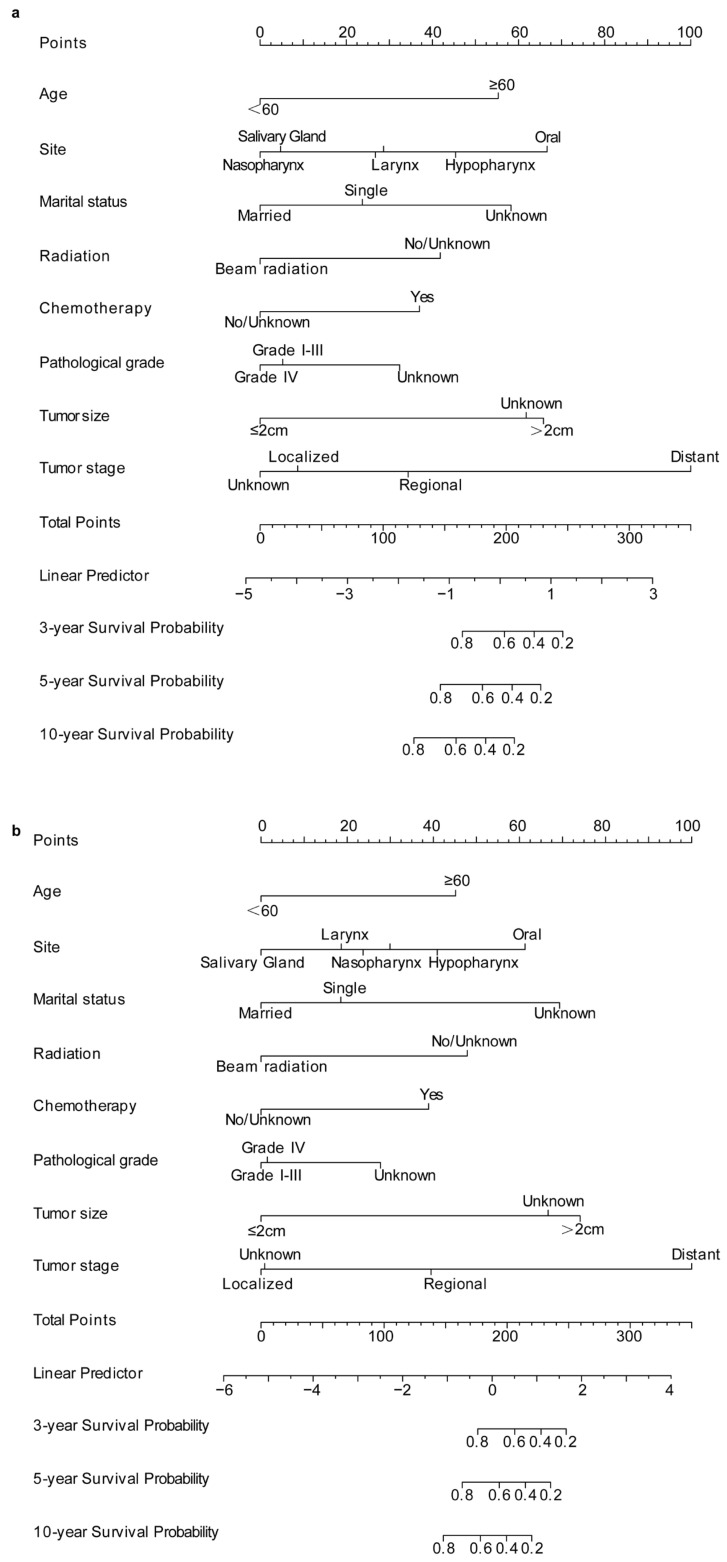
Nomograms constructed to predict 3-, 5-, and 10-year OS (**a**) and CSS (**b**) rates in patients with HNCS.

Key prognostic factors identified via the Akaike Information Criterion (AIC) were used to construct nomograms for estimating 3-, 5-, and 10-year OS and CSS for HNCS patients. Each factor in the nomogram is assigned a score on a logarithmic scale, enabling prediction of OS or CSS by summing these scores to obtain a total value, which corresponds to survival probabilities on the bottom scale (Figure 2).

The nomogram’s predictive accuracy was evaluated using the concordance index (C-index), calibration plots, and receiver operating characteristic (ROC) curves. In the training cohort, the C-index for OS was 0.757 (95% CI, 0.723–0.790), and for the validation cohort, it was 0.777 (95% CI, 0.736–0.818). For CSS, the C-index was 0.779 (95% CI, 0.744–0.815) in the training group and 0.776 (95% CI, 0.725–0.827) in the validation group, indicating high concordance between the predicted and observed outcomes.

**Figure 3:**
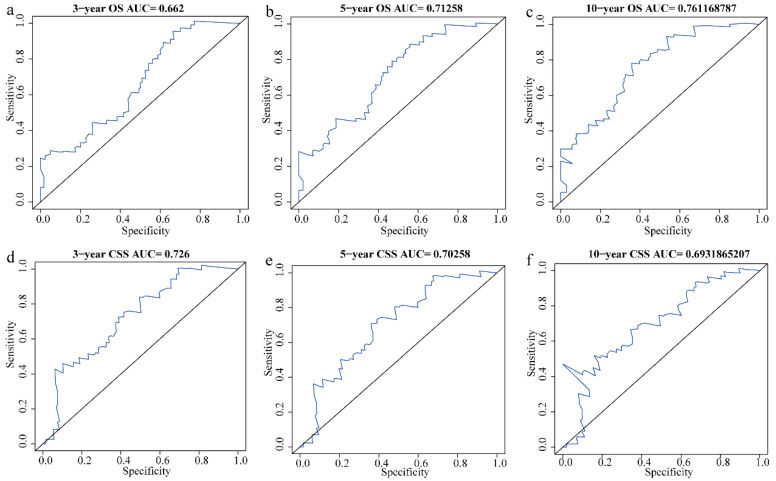
Receiver operating characteristic (ROC) curves for evaluating the predictive ability of independent prognostic factors for 3-, 5-, and 10-year OS and CSS in HNCS patients in the training cohort: (**a**) 3-year OS; (**b**) 5-year OS; (**c**) 10-year OS; (**d**) 3-year CSS; (**e**) 5-year CSS; (**f**) 10-year CSS.

**Figure 4:**
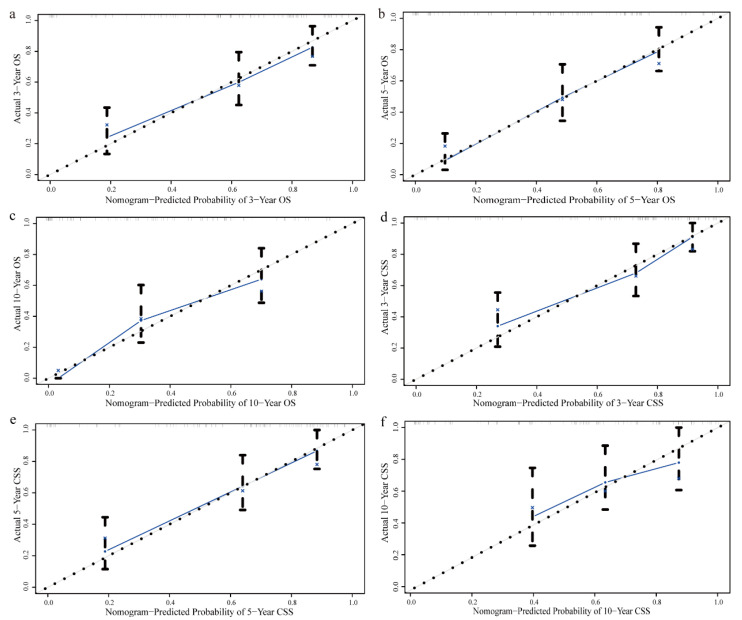
Calibration curves for the nomogram in the training group: (**a**) 3-year OS, (**b**) 5-year OS, (**c**) 10-year OS, (**d**) 3-year CSS, (**e**) 5-year CSS, and (**f**) 10-year CSS.

**Figure 5:**
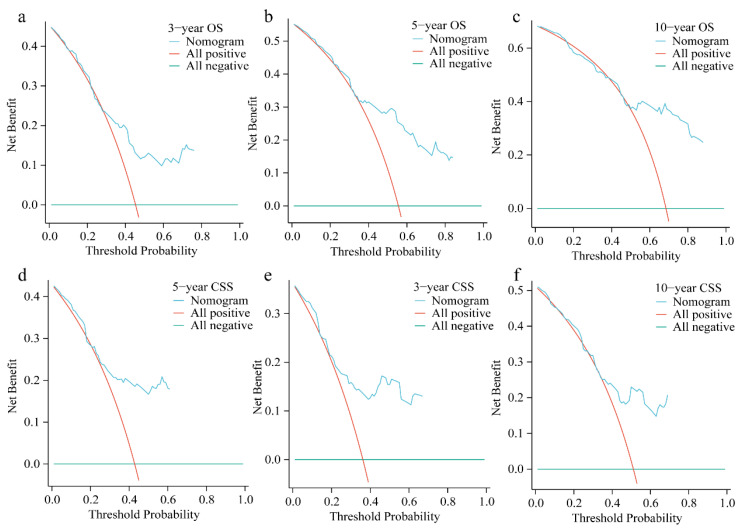
Decision curve analysis for the nomograms in the training group: (**a**) 3-year OS, (**b**) 5-year OS, (**c**) 10-year OS, (**d**) 3-year CSS, (**e**) 5-year CSS, and (**f**) 10-year CSS. The *x*-axis represents the threshold probability, and the *y*-axis represents the net benefit.

The areas under the ROC curve (AUC) for assessing the predictive accuracy of independent prognostic factors in the training cohort were 0.662, 0.713, and 0.761 for 3-, 5-, and 10-year OS, respectively, and 0.726, 0.703, and 0.693 for CSS (Figure 3). In the validation cohort, the AUC values for predicting 3-, 5-, and 10-year OS were 0.753, 0.777, and 0.829, respectively, while the AUC values for CSS were 0.718, 0.691, and 0.693 (Supplementary Figure S3). Calibration plots showed strong agreement between the predicted and actual survival probabilities (Figure 4, Supplementary Figure S4). Finally, decision curve analysis (DCA) demonstrated the clinical applicability of the nomogram models, supporting their utility in clinical decision-making (Figure 5, Supplementary Figure S5).

### 3.5. K-M Analysis

**Figure 6:**
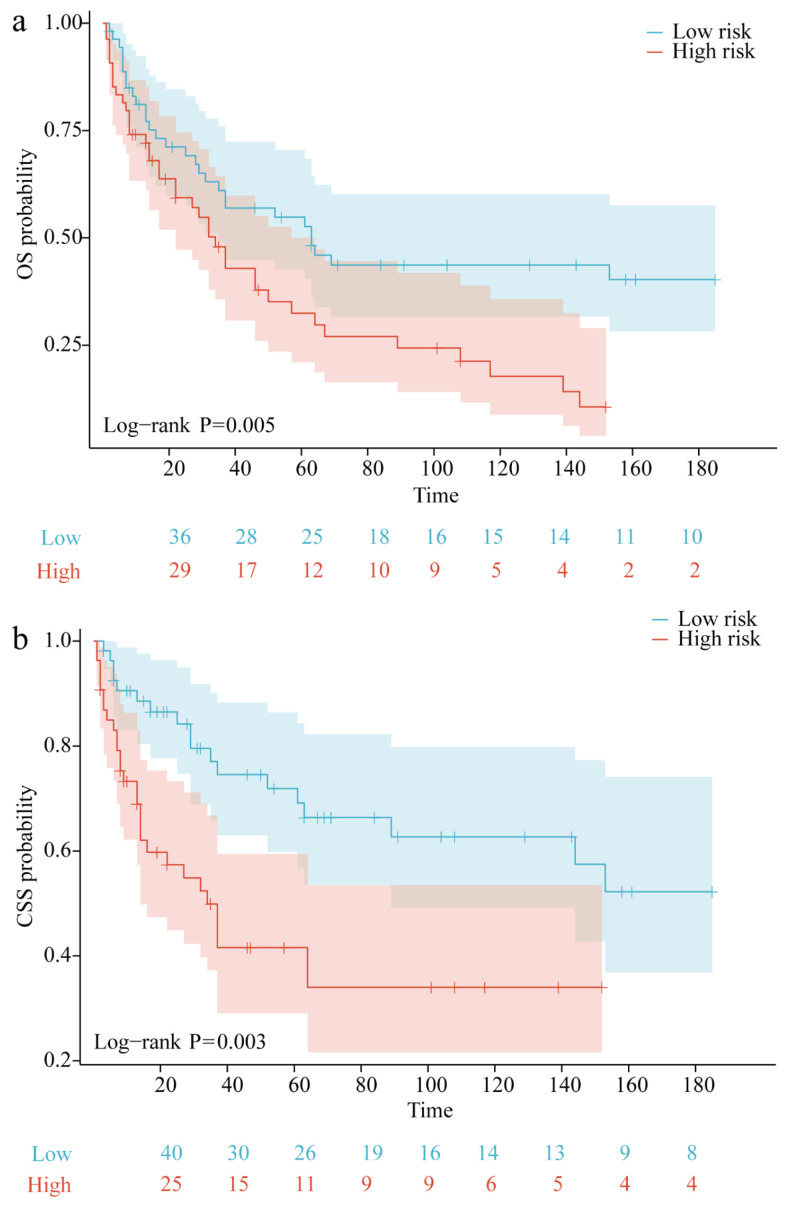
Kaplan–Meier (K-M) curves for the nomogram in the training group: (**a**) OS and (**b**) CSS.

Prognostic risk scores were calculated based on factors included in the prognostic nomogram, enabling classification of patients into high-risk and low-risk groups to evaluate the model’s feasibility and validity. Kaplan–Meier (K-M) survival curves revealed statistically significant differences in prognosis between these groups (*p* < 0.05), confirming the model’s effectiveness in identifying high-risk individuals for both overall survival (OS) and cancer-specific survival (CSS). Additionally, the K-M curves indicate that high-risk patients have markedly lower CSS and OS compared to low-risk patients (Figure 6).

## 4. Discussion

This study analyzed data from 152 HNCS patients in the SEER database (1975–2020) to develop and validate the first large-scale, population-based nomograms for predicting OS and CSS in HNCS. Based on clinicopathological and treatment data, our study addresses a gap in the literature, as previous studies on HNCS were primarily limited to case reports, systematic reviews, or small retrospective analyses lacking comprehensive examination of prognostic factors and survival outcomes [5,12,13,14,15,16,17,18,19]. For instance, Gupta et al. analyzed 66 cases of salivary carcinosarcoma in 2020, finding a 5-year OS of 37% and a CSS of 62% [13], while Patel et al. reported a 5-year CSS of 48.5% for 15 sinonasal carcinosarcoma cases [5]. However, due to their small sample sizes, these studies did not assess independent prognostic factors or develop predictive models. Our study, therefore, provides a more comprehensive analysis of prognostic factors and introduces nomograms to estimate 3-, 5-, and 10-year OS and CSS for HNCS patients.

Our analysis identified eight key prognostic variables—age, primary site, marital status, radiation therapy, chemotherapy, pathological grade, tumor size, and tumor stage—which were incorporated into the nomograms. Each variable contributes a score based on its prognostic impact, enabling clinicians to calculate a total score that corresponds to survival probabilities. This nomogram serves as a practical, visual tool for integrating multiple prognostic factors into a single, easy-to-use scoring system, aiding clinicians in individualized prognosis assessments and treatment planning. However, due to limitations within the SEER dataset, including a lack of detailed treatment information, external validation with an independent cohort is essential to confirm the model’s clinical utility across diverse populations. Future studies should focus on multicenter collaborations to collect comprehensive treatment data, such as specific chemotherapy and radiotherapy regimens, to further enhance the model’s accuracy and applicability.

The results show several important trends in HNCS. We observed a gradual increase in the average age at diagnosis over the study period, potentially reflecting demographic shifts or improvements in detection among older patients. Consistent with prior findings [5,12,13], males accounted for 63.2% of cases, and salivary gland tumors were the most frequent primary site (41.7%), followed by laryngeal (21.1%) and nasal cavity or middle ear tumors (15.1%). Survival outcomes also varied notably by primary site and disease stage. The 5-year OS rates were highest for laryngeal carcinosarcomas (57.3%) and lowest for oral cavity tumors (26.1%), indicating significant site-specific survival differences. These findings align with previous reports [5,13], underscoring the need for tailored treatment strategies based on tumor location.

Although awareness of the rarity of HNCS is increasing, standardized treatment guidelines and protocols remain lacking, and the biological behavior of HNCS is not well understood. Management of HNCS often mirrors that of head and neck squamous cell carcinoma, given the limited data specific to this tumor type [1]. Complete surgical excision with adequate margins is generally regarded as the primary treatment approach, with adjustments based on tumor extent, lymph node involvement, and metastasis [13]. For example, laryngeal and hypopharyngeal carcinosarcomas may be managed through local excision, partial excision, or total laryngectomy, while lesions in other head and neck regions may require more extensive resection [20].

The efficacy of radiotherapy and chemotherapy in HNCS remains controversial due to insufficient data. Zhang Y et al. recommend adjuvant radiotherapy following lymph node dissection for cases with lymph node metastasis and suggest it as an option for patients who are inoperable or have unresectable lesions [14]. Conversely, Zhang M et al., in a review of 13 cases of laryngeal carcinosarcoma, found that the sarcoma component showed resistance to radiation, concluding that postoperative radiotherapy may not be beneficial for controlling local recurrence or metastasis in these cases [1]. In our study, 83.6% of patients underwent surgery, 69.1% received radiotherapy, and only 21.7% received chemotherapy. While surgery was significantly associated with CSS in univariate analysis, it did not maintain significance in multivariate analysis, and neither radiotherapy nor chemotherapy showed a significant correlation with prognosis.

The SEER database provides the largest accessible dataset for rare tumors like HNCS, yet it has notable limitations. A significant limitation of this study is the lack of detailed information on chemotherapy drugs and radiotherapy regimens in SEER, which restricts our ability to assess the effectiveness of specific treatment modalities for HNCS patients. Without granular data on the types of chemotherapy agents used, dosages, or radiotherapy techniques and dosimetry, it is challenging to evaluate the efficacy of these treatments under different clinical conditions. This lack of treatment detail means that our findings regarding the non-significant impact of these treatments on survival outcomes should be interpreted with caution. Furthermore, our study utilizes SEER records spanning several decades (1975–2020), introducing variability due to evolving diagnostic and treatment standards. Over this period, advances in surgical techniques, radiation therapy, and chemotherapy protocols likely influenced survival outcomes, potentially biasing results based on when patients were treated. Earlier cases may have received less advanced treatments, while recent improvements in multimodal approaches may have provided survival advantages that are not directly comparable to outcomes from earlier periods. This variability may be particularly pronounced for rare conditions like HNCS, where treatment protocols are often adapted from other head and neck cancers, leading to inconsistencies across institutions and time periods.

To enhance data reliability, we excluded patients lacking complete clinical demographics or follow-up data. While this criterion ensures a more reliable dataset, it may also introduce selection bias, as patients with incomplete data could represent certain demographics, such as those from lower socioeconomic backgrounds, who may have limited access to healthcare resources or face barriers to consistent follow-up. This potential underrepresentation of specific populations might limit the generalizability of our findings, as these patients could experience different survival outcomes due to disparities in treatment access or healthcare quality.

To mitigate potential bias and improve the robustness of our analysis, we applied a randomization process to divide the remaining patients into training and validation cohorts, ensuring balanced demographic and clinical characteristics between groups. Future research could address data incompleteness more comprehensively, potentially using imputation methods or the use of complementary data sources that may capture a broader patient demographic. Additionally, studies that explore socioeconomic factors and healthcare access as independent variables could provide valuable insights into their impact on survival outcomes in HNCS.

While our nomograms were internally validated using a random division of the study cohort into training and validation groups, this internal validation alone may not fully establish the models’ generalizability or clinical utility. External validation with an independent cohort is essential to confirm the robustness and applicability of these prognostic tools across diverse populations and clinical settings. Future studies should aim to validate these nomograms in external cohorts through multicenter collaborations or prospective studies. Testing the models in varied real-world settings would strengthen their reliability and ensure adaptability to different clinical environments, ultimately facilitating broader adoption in clinical practice.

Information bias, arising from potential inaccuracies in data collection or recording within large databases like SEER, presents a concern in this study. To reduce information bias, we applied strict inclusion criteria and conducted a multivariable analysis to account for a wide range of prognostic factors. Randomization further helped balance demographic and clinical characteristics between the training and validation cohorts, minimizing systematic differences. However, future research could mitigate information bias more effectively by incorporating prospective data collection or supplementary Data sources to verify and enrich the models.

In summary, this study provides a comprehensive analysis of prognostic factors for HNCS using SEER data, leading to the development of the first predictive nomograms for OS and CSS in HNCS patients. Although constrained by limitations in treatment data and the need for external validation, our findings contribute to a more nuanced understanding of HNCS and offer a potential tool for clinical prognosis. Future research should focus on external validation and expanded data collection to establish robust, standardized guidelines for managing this rare malignancy.

## Figures and Tables

**Table 1:** Baseline characteristics of HNCS patients included in the study.

Variables	Training	Validation	Overall	χ^2^	*p* Value
	(*N* = 108)	(*N* = 44)	(*N* = 152)		
Age				0.0033191	0.9541
<60	44 (40.7%)	17 (38.6%)	61 (40.1%)		
≥60	64 (59.3%)	27 (61.4%)	91 (59.9%)		
Sex				7.40 × 10^31^	1
Female	40 (37.0%)	16 (36.4%)	56 (36.8%)		
Male	68 (63.0%)	28 (63.6%)	96 (63.2%)		
Race				0.32668	0.8493
Black	14 (13.0%)	6 (13.6%)	20 (13.2%)		
Other	5 (4.6%)	3 (6.8%)	8 (5.3%)		
White	89 (82.4%)	35 (79.5%)	124 (81.6%)		
Site				2.3591	0.7976
Hypopharynx	4 (3.7%)	1 (2.3%)	5 (3.3%)		
Larynx	25 (23.1%)	7 (15.9%)	32 (21.1%)		
Nasopharynx	3 (2.8%)	1 (2.3%)	4 (2.6%)		
Nose, Nasal Cavity, and Middle Ear	17 (15.7%)	6 (13.6%)	23 (15.1%)		
Oral	14 (13.0%)	9 (20.5%)	23 (15.1%)		
Salivary Gland	45 (41.7%)	20 (45.5%)	65 (42.8%)		
Marital Status at Diagnosis				2.429	0.2969
Married	51 (47.2%)	20 (45.5%)	71 (46.7%)		
Single	55 (50.9%)	21 (47.7%)	76 (50.0%)		
Unknown	2 (1.9%)	3 (6.8%)	5 (3.3%)		
Surgery				0.016119	0.899
Not Performed	17 (15.7%)	8 (18.2%)	25 (16.4%)		
Performed	91 (84.3%)	36 (81.8%)	127 (83.6%)		
Radiation				0.18294	0.6689
Beam radiation	73 (67.6%)	32 (72.7%)	105 (69.1%)		
No/Unknown	35 (32.4%)	12 (27.3%)	47 (30.9%)		
Chemotherapy				0.79289	0.3732
No/Unknown	82 (75.9%)	37 (84.1%)	119 (78.3%)		
Yes	26 (24.1%)	7 (15.9%)	33 (21.7%)		
Surgery and Radiotherapy Sequence				0.000356	0.9849
Not/Unknown	51 (47.2%)	20 (45.5%)	71 (46.7%)		
Radiation after Surgery	57 (52.8%)	24 (54.5%)	81 (53.3%)		
Regional Nodes				0.37088	0.8307
All Nodes Examined Negative	26 (24.1%)	12 (27.3%)	38 (25.0%)		
No Nodes Examined/Unknown	69 (63.9%)	28 (63.6%)	97 (63.8%)		
Regional Nodes Positive	13 (12.0%)	4 (9.1%)	17 (11.2%)		
Pathological Grade				0.29568	0.8626
Grade I-III	25 (23.1%)	12 (27.3%)	37 (24.3%)		
Grade IV	24 (22.2%)	9 (20.5%)	33 (21.7%)		
Unknown	59 (54.6%)	23 (52.3%)	82 (53.9%)		
Tumor Size				0.73793	0.6915
>2 cm	68 (63.0%)	28 (63.6%)	96 (63.2%)		
≤2 cm	12 (11.1%)	3 (6.8%)	15 (9.9%)		
Unknown	28 (25.9%)	13 (29.5%)	41 (27.0%)		
Tumor Stage				6.5512	0.08767
Distant	13 (12.0%)	4 (9.1%)	17 (11.2%)		
Localized	31 (28.7%)	21 (47.7%)	52 (34.2%)		
Regional	40 (37.0%)	15 (34.1%)	55 (36.2%)		
Unknown	24 (22.2%)	4 (9.1%)	28 (18.4%)		

**Table 2:** Univariate and multivariate Cox regression analyses for OS in HNCS patients from the training cohort.

Characteristics	Total (*N*)	HR (95% CI) Univariate Analysis	*p* Value Univariate Analysis	HR (95% CI) Multivariate Analysis	*p* Value Multivariate Analysis
Age	108				
<60	44	Reference		Reference	
≥60	64	2.539 (1.497–4.306)	<0.001	2.231 (1.239–4.016)	0.007
Sex	108				
Female	40	Reference			
Male	68	0.966 (0.596–1.567)	0.889		
Race	108				
White	89	Reference			
Black	14	1.208 (0.593–2.460)	0.603		
Other	5	0.806 (0.252–2.583)	0.717		
Site	108				
Oral	14	Reference		Reference	
Salivary Gland	45	0.336 (0.171–0.662)	0.002	0.324 (0.151–0.697)	0.004
Larynx	25	0.503 (0.249–1.013)	0.054	0.440 (0.197–0.985)	0.046
Nose, Nasal Cavity, and Middle Ear	17	0.332 (0.143–0.770)	0.010	0.544 (0.208–1.423)	0.215
Nasopharynx	3	0.112 (0.014–0.894)	0.039	0.244 (0.025–2.338)	0.221
Hypopharynx	4	0.752 (0.247–2.288)	0.616	0.878 (0.265–2.905)	0.831
Marital status	108				
Single	55	Reference		Reference	
Married	51	0.575 (0.353–0.938)	0.027	0.559 (0.314–0.995)	0.048
Unknown	2	2.405 (0.576–10.040)	0.229	2.998 (0.423–21.231)	0.272
Surgery	108				
Performed	91	Reference		Reference	
Not Performed	17	1.789 (0.954–3.357)	0.070	1.361 (0.574–3.223)	0.484
Radiation	108				
Beam Radiation	73	Reference		Reference	
No/Unknown	35	1.538 (0.936–2.527)	0.089	1.504 (0.803–2.818)	0.203
Chemotherapy	108				
Yes	26	Reference			
No/Unknown	82	1.133 (0.639–2.009)	0.669		
Surgery and Radiotherapy Sequence	108				
Radiation after Surgery	57	Reference			
Not/Unknown	51	1.281 (0.799–2.052)	0.304		
Regional Nodes	108				
All Nodes Examined Negative	26	Reference		Reference	
Regional Nodes Positive	13	1.969 (0.818–4.739)	0.131	1.649 (0.532–5.115)	0.386
No Nodes Examined/Unknown	69	1.884 (0.954–3.722)	0.068	1.261 (0.510–3.119)	0.615
Pathological Grade	108				
Grade I-III	25	Reference		Reference	
Unknown	59	1.783 (0.980–3.246)	0.058	1.842 (0.891–3.807)	0.099
Grade IV	24	1.074 (0.524–2.202)	0.845	0.858 (0.359–2.053)	0.732
Tumor Size	108				
≤2 cm	12	Reference			
>2 cm	68	2.136 (0.846–5.392)	0.108		
Unknown	28	1.962 (0.735–5.237)	0.179		
Tumor Stage	108				
Regional	40	Reference		Reference	
Localized	31	0.853 (0.487–1.491)	0.576	0.747 (0.363–1.536)	0.427
Distant	13	2.548 (1.284–5.058)	0.008	3.347 (1.498–7.481)	0.003
Unknown	24	0.541 (0.235–1.247)	0.149	0.474 (0.160–1.400)	0.176

**Table 3:** Univariate and multivariate Cox regression analyses for CSS in HNCS patients from the training cohort.

Characteristics	Total (*N*)	HR (95% CI) Univariate Analysis	*p* Value Univariate Analysis	HR (95% CI) Multivariate Analysis	*p* Value Multivariate Analysis
Age	108				
<60	44	Reference		Reference	
≥60	64	1.688 (0.923–3.086)	0.089	1.645 (0.832–3.252)	0.152
Sex	108				
Female	40	Reference			
Male	68	0.999 (0.555–1.799)	0.997		
Race	108				
White	89	Reference			
Black	14	1.253 (0.530–2.965)	0.608		
Other	5	0.831 (0.200–3.453)	0.799		
Site	108				
Oral	14	Reference		Reference	
Salivary Gland	45	0.324 (0.143–0.733)	0.007	0.260 (0.103–0.653)	0.004
Larynx	25	0.446 (0.188–1.060)	0.068	0.419 (0.153–1.151)	0.092
Nose, Nasal Cavity, and Middle Ear	17	0.443 (0.174–1.126)	0.087	0.527 (0.177–1.570)	0.250
Nasopharynx	3	0.245 (0.031–1.950)	0.184	0.236 (0.025–2.256)	0.210
Hypopharynx	4	0.772 (0.212–2.808)	0.694	0.812 (0.209–3.156)	0.763
Marital Status	108				
Single	55	Reference		Reference	
Married	51	0.635 (0.351–1.149)	0.133	0.637 (0.322–1.260)	0.195
Unknown	2	3.293 (0.775–13.982)	0.106	1.769 (0.177–17.738)	0.627
Surgery	108				
Performed	91	Reference		Reference	
Not Performed	17	2.463 (1.243–4.878)	0.010	2.923 (0.699–12.229)	0.142
Radiation	108				
Beam Radiation	73	Reference		Reference	
No/Unknown	35	1.648 (0.907–2.993)	0.101	2.151 (0.505–9.155)	0.300
Chemotherapy	108				
Yes	26	Reference			
No/Unknown	82	0.748 (0.400–1.399)	0.363		
Surgery and Radiotherapy Sequence	108				
Radiation after Surgery	57	Reference		Reference	
Not/Unknown	51	1.501 (0.845–2.665)	0.166	0.581 (0.118–2.857)	0.504
Regional Nodes	108				
All Nodes Examined Negative	26	Reference		Reference	
Regional Nodes Positive	13	2.634 (0.912–7.611)	0.074	1.632 (0.459–5.811)	0.449
No Nodes Examined/Unknown	69	2.091 (0.876–4.992)	0.097	1.130 (0.378–3.384)	0.827
Pathological Grade	108				
Grade I-III	25	Reference			
Unknown	59	1.469 (0.719–3.003)	0.292		
Grade IV	24	1.098 (0.476–2.533)	0.827		
Tumor Size	108				
≤2 cm	12	Reference			
>2 cm	68	2.470 (0.754–8.089)	0.135		
Unknown	28	2.184 (0.622–7.675)	0.223		
Tumor Stage	108				
Regional	40	Reference		Reference	
Localized	31	0.470 (0.217–1.017)	0.055	0.547 (0.217–1.377)	0.200
Distant	13	2.575 (1.206–5.497)	0.015	3.569 (1.397–9.114)	0.008
Unknown	24	0.509 (0.193–1.343)	0.172	0.490 (0.131–1.836)	0.290

## Data Availability

The data used in this study are available from the Surveillance, Epidemiology, and End Results Program (SEER) database (https://seer.cancer.gov/data/access.html (accessed on 23 March 2024)).

## Supplementary Materials

The following supporting information can be downloaded at: https://www.mdpi.com/article/10.3390/biomedicines12112556/s1, Figure S1: Flowchart of the screening and selection process for HNCS patients from the SEER database; Figure S2: Trend in age at diagnosis for HNCS patients from 1975 to 2020; Figure S3: ROC curves for evaluating the predictive ability of independent prognostic factors for 3-, 5-, and 10-year OS and CSS in HNCS patients in the validation cohort; Figure S4: Calibration curves for the nomogram in the validation group; Figure S5: Decision curve analysis for the nomograms in the validation group.
