# Supplementary material for: The Demographic and Clinical Characteristics, Prognostic Factors, and Survival Outcomes of Head and Neck Carcinosarcoma: A SEER Database Analysis
Source: Biomedicines. 2024 Nov 8;12(11):2556. doi: 10.3390/biomedicines12112556 (PMC11591622; doi:10.3390/biomedicines12112556)
Supplement: Supplementary file 1 [file biomedicines-12-02556-s001.zip › biomedicines-3280866-supplementary.pdf]

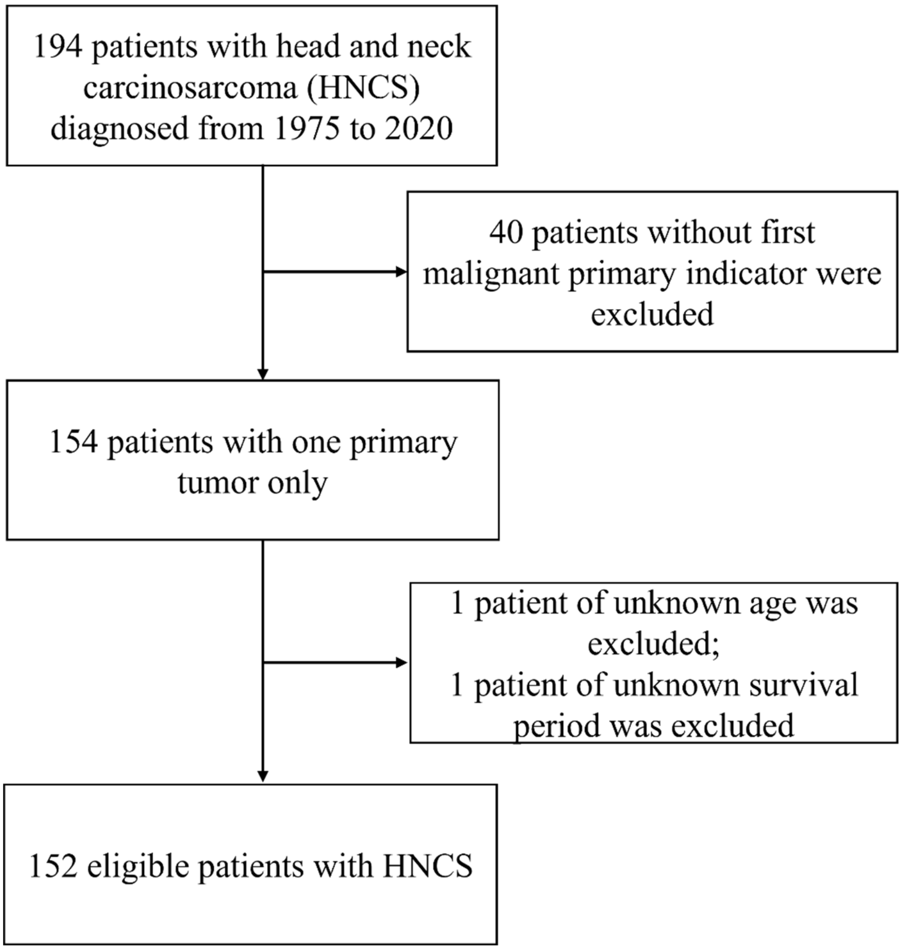

Supplementary Figure S1. Flowchart of the screening and selection process for HNCS patients from the SEER database.

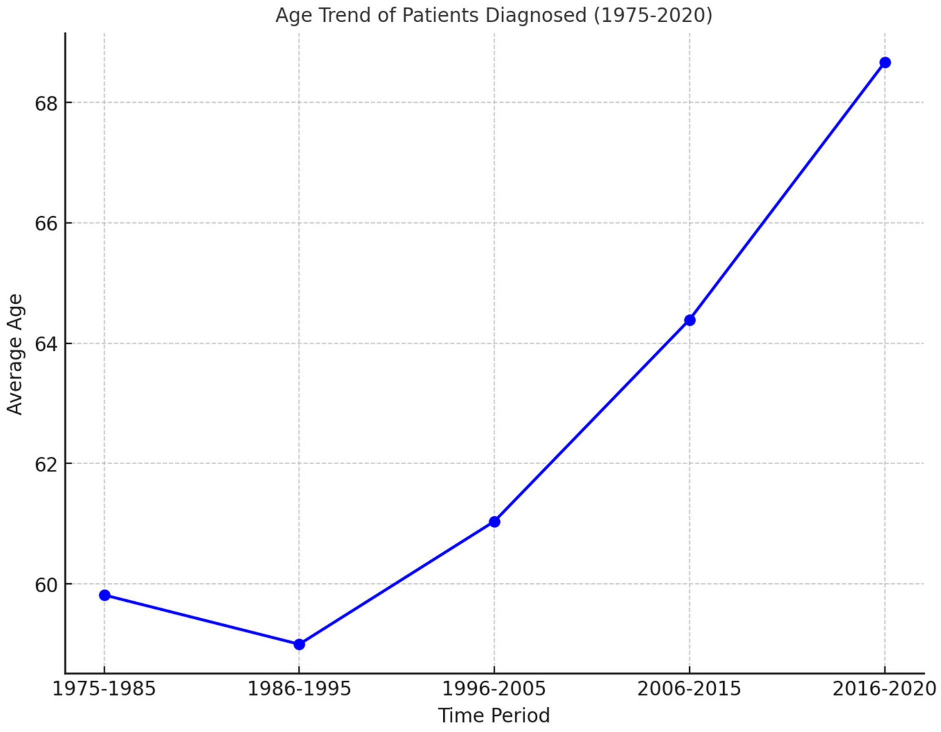

Supplementary Figure S2. Trend in age at diagnosis for HNCS patients from 1975 to 2020.

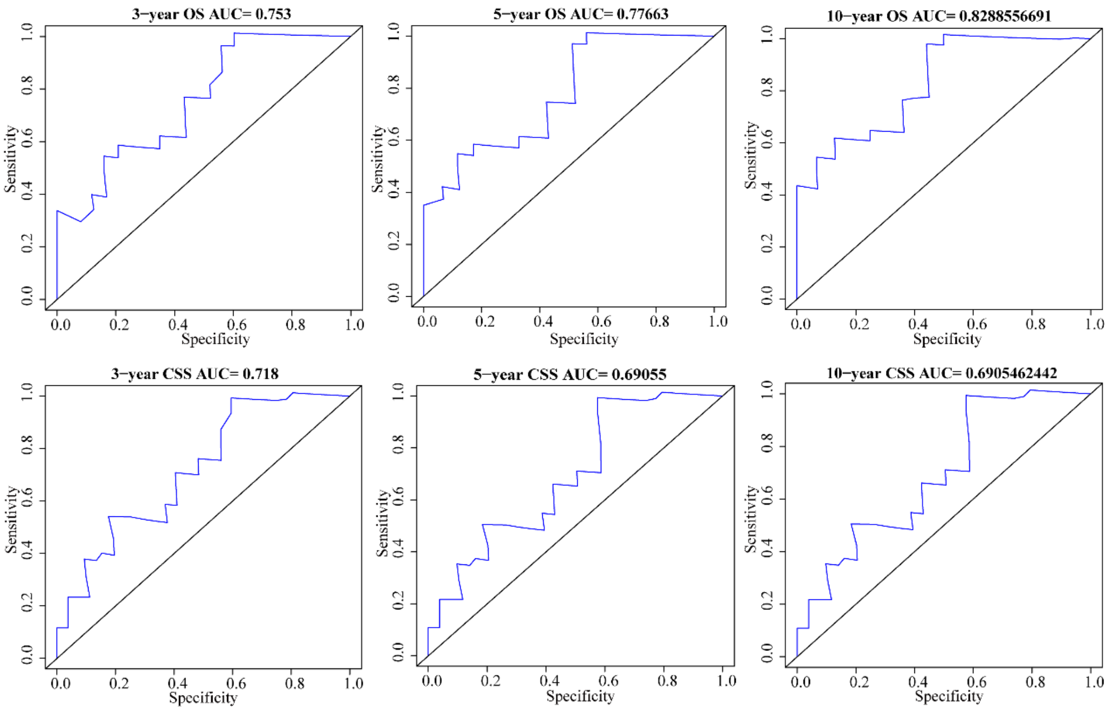

Supplementary Figure S3. ROC curves for evaluating the predictive ability of independent prognostic factors for 3-, 5-, and 10-year OS and CSS in HNCS patients in the validation cohort: (a) 3-year OS; (b) 5-year OS; (c) 10-year OS; (d) 3-year CSS; (e) 5-year CSS; (f) 10-year CSS.

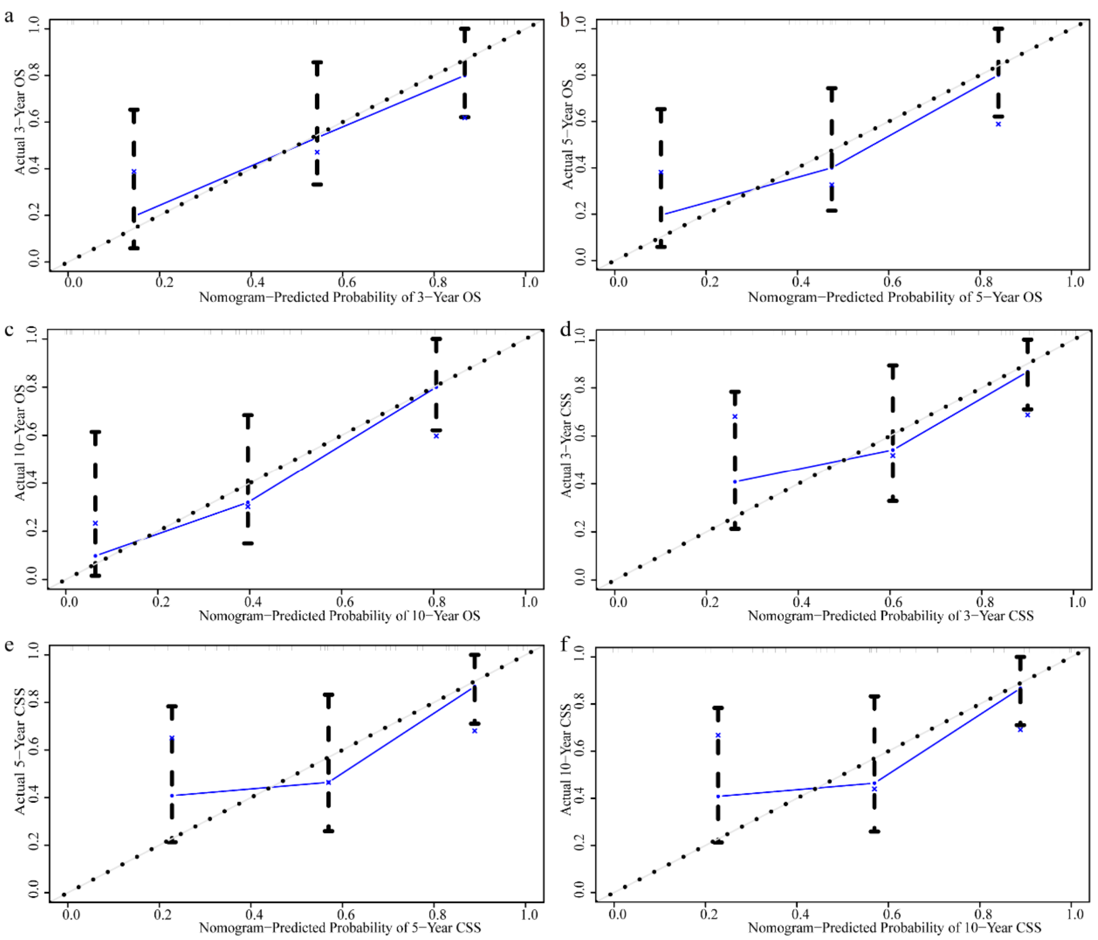

Supplementary Figure S4. Calibration curves for the nomogram in the validation group: (a) 3-year OS, (b) 5-year OS, (c) 10-year OS, (d) 3-year CSS, (e) 5-year CSS, and (f) 10-year CSS.

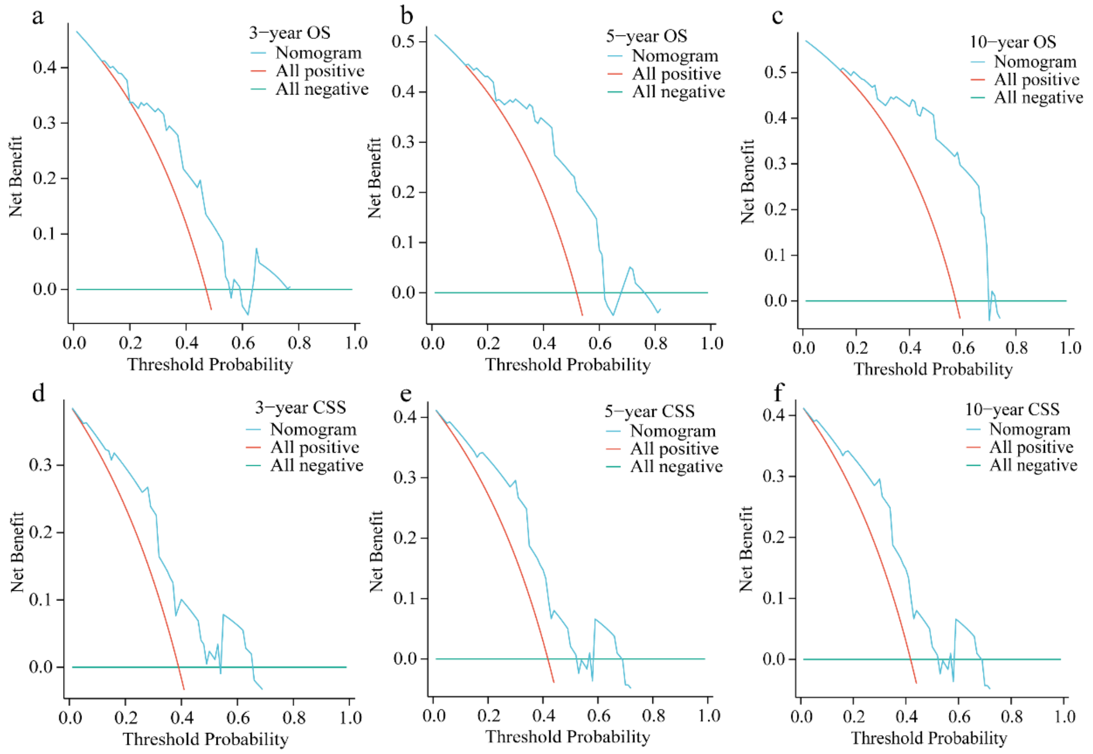

Supplementary Figure S5. Decision curve analysis for the nomograms in the validation group: (a) 3-year OS, (b) 5-year OS, (c) 10-year OS, (d) 3-year CSS, (e) 5-year CSS, and (f) 10-year CSS. The x-axis represents the threshold probability, and the y-axis represents the net benefit.
